# Particulate emissions from L-Category vehicles towards Euro 5

**DOI:** 10.1016/j.envres.2019.109071

**Published:** 2020-03

**Authors:** A. Kontses, L. Ntziachristos, A.A. Zardini, G. Papadopoulos, B. Giechaskiel

**Affiliations:** a Laboratory of Applied Thermodynamics, Aristotle University of Thessaloniki, P.O. Box 458, GR 54124, Thessaloniki, Greece; b European Commission Joint Research Center, Directorate for Energy, Transport and Climate, Sustainable Transport Unit, 21027, Ispra (VA), Italy; c Emisia S.A., Antoni Tritsi 21, PO Box 8138, GR-57001, Thessaloniki, Greece

**Keywords:** Particulate emissions, Particle number, L-category, Mopeds, Motorcycles, Quads, Minicars, Euro 5

## Abstract

The current experimental study presents particulate emissions from 30 Euro 1-4 L-category vehicles (i.e. 2-, 3- and 4-wheelers such as mopeds, motorcycles, quads and minicars, registered in Europe between 2009 and 2016) tested on a chassis dynamometer. The objectives were to identify those sub-categories with high emissions, to assess whether the measures prescribed in the Euro 5 legislation will effectively control particulate emissions and finally to investigate the need for additional measures. The results showed that 2-stroke (2S) mopeds and diesel minicars comprised the vehicles with the highest particulate mass (PM) and solid particle number above 23 nm (SPN23) emissions (up to 64 mg/km and 4.5 × 10^13^ km^−1^, respectively). It is uncertain whether the installation of diesel particulate filters (DPF) is a cost-effective measure for diesel mini-cars in order to comply with Euro 5 standard, while advanced emission controls will be required for 2S mopeds, if such vehicles remain competitive for Euro 5. Regarding 4-stroke mopeds, motorcycles and quads, PM emissions were one order of magnitude lower than 2S ones and already below the Euro 5 limit. Nevertheless, SPN23 emissions from these sub-categories were up to 5 times higher than the Euro 6 passenger cars limit (6 × 10^11^ km^−1^). Even recent Euro 4 motorcycles exceeded this limit by up to 3 times. These results indicate that L-category vehicles are a significant contributor to vehicular particulate emissions and should be further monitored during and after the introduction of the Euro 5 step. Moreover, including SPN in the range 10–23 nm increases emission levels by up to 2.4 times compared to SPN23, while volatile and semi-volatile particle numbers were even higher. Finally, cold engine operation was found to be a significant contributor on SPN23 emissions, especially for vehicles with lower overall emission levels. These results indicate that a specific particle number limit may be required for L-category to align emissions with passenger cars.

## Highlights

• L-category is becoming a major contributor to vehicular particulate emissions.
• 2-Stroke mopeds and diesel minicars comprise the highest emitters.
• High particle number emissions are observed even if particulate mass is low.
• Particulate emissions of latest technology vehicles much higher than passenger cars.
• A revision of particulate emissions regulation for L-category shall be investigated.

## Graphical abstract

**Figure:**
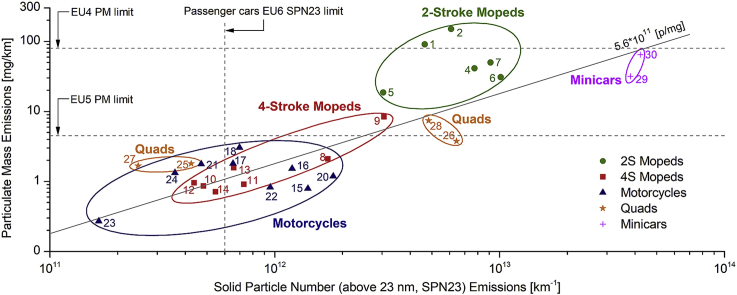

## Abbreviations

2S: 2-Stroke
2WC: 2-Way Catalyst
3WC: 3-Way Catalyst
4S: 4-Stroke
APC: AVL Particle Counter
CI: Compression Ignition
CS: Catalytic Stripper
CV: Coefficient of Variation
CPC: Condensation Particle Counter
CVS: Constant Volume Sampling
DPF: Diesel Particulate Filter
ECE: Economic Commission for Europe
EEA: European Environment Agency
EEPS: Engine Exhaust Particle Sizer
EU: European Union
GDI: Gasoline Direct Injection
GMD: Geometric Mean Diameter
GPF: Gasoline Particulate Filter
HC: Hydrocarbons
HEPA: High-efficiency Particulate Filter
JRC: Joint Research Center
PAH: Polycyclic Aromatic Hydrocarbons
PEMS: Portable Emissions Measurement System
PFI: Port Fuel Injection
PM: Particulate Mass
PMP: Particle Measurement Programme
PM_2.5_: Fine Particulate Mass (sub-2.5 μm)
PTFE: Polytetrafluoroethylene
SPN: Solid Particle Number
SPN10: Solid Particle Number with a size cut-off at 10 nm
SPN23: Solid Particle Number with a size cut-off at 23 nm
THC: Total Hydrocarbons
TPN10: Total Particle Number with a size cut-off at 10 nm
UNECE: United Nations Economic Commission for Europe
WMTC: Worldwide harmonized Motorcycle Test Cycle

## 1 Introduction

Particulate matter is a major concern for urban air quality nowadays, causing more than 0.4 million premature deaths each year in Europe (EEA, 2018). Road transport has been a major source of anthropogenic particulate matter emissions over the last decades (Morawska et al., 2008; Borsós et al., 2012; EEA, 2018), being currently responsible for approximately 11% of fine particulate mass (PM_2.5_) emissions in Europe (EEA, 2018). Since the beginning of the 1990's much attention has been given to particulate emissions of diesel light and heavy-duty vehicles. Thus, as a first step the European Union (EU) introduced a particulate mass (PM) emissions limit for these vehicles, with the enforcement of Euro 1 (light-duty vehicles) and Euro I (heavy-duty vehicles) emission standards (EU, 1991a, 1991b, 1993). Several regulation amendments and revisions were implemented since then, pushing the limits to significantly lower values. Road vehicles were also found to be responsible for up to 90% of particle number emissions in busy roads (Kumar et al., 2010), thus in 2011 (Euro 5b) a solid particle number (SPN) limit with a size cut-off at 23 nm (SPN23) was brought into force for light-duty diesel vehicles and two years later for heavy-duty ones (EU, 2007, 2008, 2011). High SPN23 emissions were also observed from gasoline light-duty vehicles equipped with a direct injection (GDI) engine (Giechaskiel et al., 2014), thus PM and SPN23 limits were implemented in 2009 and 2014, respectively. EU is currently working on introducing a lower size cut-off for PN emissions of 10 nm (UNECE, 2019).

This regulatory framework brought significant improvements in emissions control and resulted to a 50% decrease of PM_2.5_ emissions from road vehicles in the period 2000–2016 (EEA, 2018). The most important step in vehicle emissions control was the introduction of diesel particulate filters (DPF) and gasoline particulate filters (GPF) which can capture the majority of particles emitted (Mamakos et al., 2013; Fiebig et al., 2014; Jang et al., 2018; Giechaskiel et al., 2019a). Consequently, the relative contribution of light and heavy-duty vehicles on anthropogenic PM emissions has gradually decreased, with L-category vehicles, comprising 2- and 3- wheelers (e.g. mopeds and motorcycles) and small quadricycles (e.g. quads and minicars) as shown in Table 1, coming more into focus (Giechaskiel et al., 2015). Based on the data reported in the EU-28 inventory report under the UNECE Convention for Long-Range Transboundary Air Pollution (EMEP/EEA, 2017) the average contribution of mopeds and motorcycles to road transport exhaust PM_2.5_ emissions over the period 2000–2017 was 3.2%. Grana et al. (2017) demonstrated that in Rome's center, motorbikes contribution to total ultrafine particle emissions can reach 30%, while for passenger cars this was approximately 16%. In addition, a study performed by Platt et al. (2014) demonstrated that two-stroke scooters comprise a significant air pollution source in modern cities. Based on a study performed by Ntziachristos et al. (2008), the contribution of L-category vehicles to total road transport PM emissions in urban areas is expected to reach 20%.

**Table 1:**
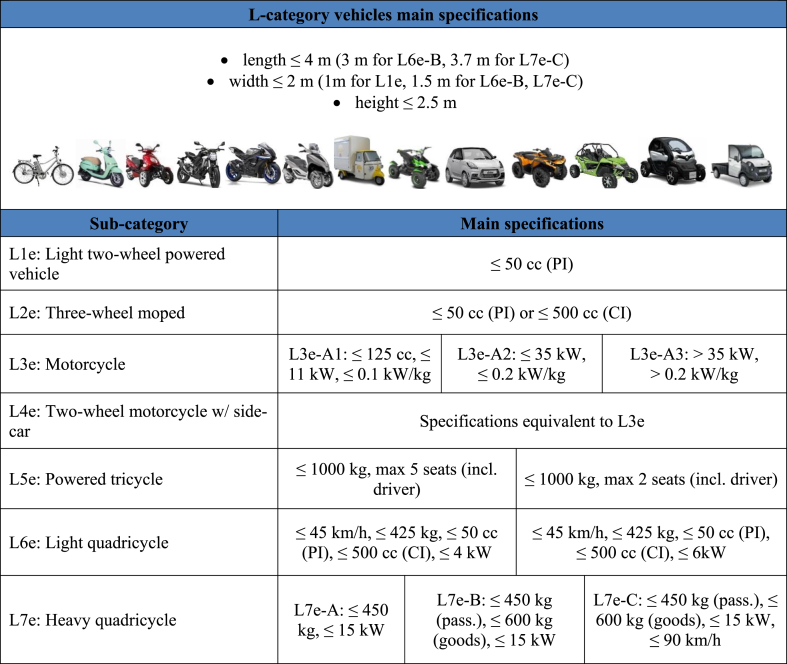
Main specifications and example vehicles of L-sub-categories. Notes: PI: Positive Ignition, CI: Compression Ignition.

Despite the increasing contribution of L-category vehicles, a PM limit for such vehicles was not introduced until 2016–2018 (depending on sub-category and new or existing vehicle type), when EU applied a lenient PM limit of 80 mg/km on Euro 4 vehicles (excluding mopeds) equipped with compression ignition (CI) engines. In 2020–2021, a more stringent limit of 4.5 mg/km, the same as the passenger cars, is to be applied for Euro 5 CI and GDI engines of all sub-categories. Table 2 presents the applicable Euro 4 and 5 PM limits for each sub-category. It should be noted that for some sub-categories (minicars, three-wheel mopeds for utility purposes, and enduro and trial motorcycles) an extra lead time of 2 years is foreseen in Euro 5 step (EU, 2018a, 2018b).

**Table 2:** Applicable PM limits in Euro 4 and 5 steps for the different L-sub-categories.

Sub-category	Applicable PM limit	
	Euro 4	Euro 5
L1e, L2e	–	4.5 mg/km applicable to: • CI engines • GDI engines • CI or GDI Hybrid powertrains
L3e – L7e	80 mg/km applicable to: • CI engines • CI Hybrid powertrains	

With this background, the first policy-related question is whether CI and GDI engines on L-category vehicles are the only significant sources of particulate emissions or whether other engine technologies should be also considered. Two-stroke (2S) mopeds, for instance, are notorious high PM and SPN emission vehicles (Ntziachristos and Galassi, 2014) but they do not necessarily fall under the GDI definition. A study performed by Rijkeboer et al. (2004) revealed that PM emissions from 2S L-category vehicles can be at the same or even higher levels compared to non-DPF diesel passenger cars. This is also confirmed by a more recent study performed by Arjan et al. (2017), who found that SPN23 emissions from Euro 2 2S mopeds were in the range 1.2–4.7 × 10^13^ km^−1^ and PM emissions varied between 57 and 262 mg/km. Apart from 2S mopeds, high SPN emissions were also detected in other sub-categories, such as the diesel 3-wheelers (in the order of 10^14^ km^−1^, Giechaskiel et al., 2015) gasoline quads and motorcycles (above 10^13^ km^−1^ and 10^12^ km^−1^ respectively, Favre et al., 2009; Giechaskiel et al., 2015). These gasoline vehicles were equipped with port fuel injection (PFI) or carburettor engines, hence they are not covered by Euro 4 and 5 PM limits. Thus, the extension of Euro 5 PM limit to these engine types may be required. Even if this happens, it is not clear if further measures, such as the introduction of a SPN23 limit, will be required in order to eliminate vehicles with high particulate emissions from the market.

The scope of the current study, which builds on the original performed to assess the cost-benefit of introducing Euro 5 for L-category (Ntziachristos et al., 2017), is to present particulate emission levels from 30 L-category vehicles. This is in order to provide the required technical background to any decisions for more stringent particulate emissions control for L-category vehicles. Compared to the above-mentioned effect study, the current work covers a wider range of L-category vehicles with additional measurements and provides a better insight on sub-category specific emissions. The studied vehicles cover most L-sub-categories (i.e. mopeds, motorcycles, quads and minicars) and are equipped with different engine and fuel systems, namely 2S and 4-stroke (4S) gasoline engines with carburettor and port fuel injection as well as diesel engines.

As a first step, the correlation between PM and SPN23 emissions is evaluated in order to investigate if high emission vehicles can be effectively detected by the Euro 5 PM limits or if a specific the introduction of a SPN limit may be required to do so. In addition, sub-23 nm and volatile particulate emissions are assessed to investigate whether these should also be taken into account in future regulations. Cold engine start can be a major contributor on particulate emissions especially in small low-mass engines that cool down fast; therefore, the cold start effect on particulate emissions is separately evaluated for each vehicle and sub-category. Evaporation losses from L-category vehicles may contribute to the formation of secondary pollutants in the atmosphere, including secondary organic aerosol (Chen et al., 2019), but this has not been within the focus of the current work.

## 2 Methodology

### 2.1 Facility

**Fig. 1:**
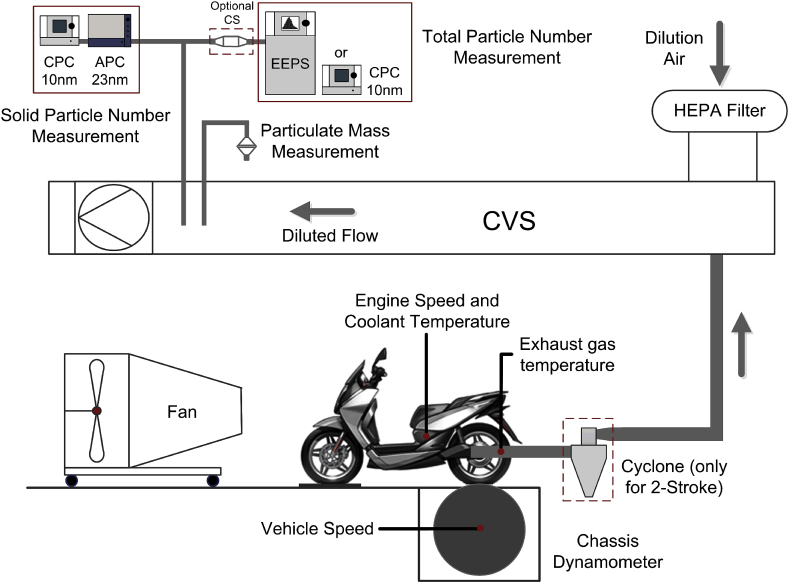
Experimental setup for particulate emissions measurement. Tested vehicle, chassis dynamometer, sampling and dilution system, and the recorded signals used in this study are presented.

Vehicles were tested in the emission test cells for 2-wheelers (mopeds and motorcycles) and small quadricycles of the Vehicles Emissions Laboratories (VELA) at the European Commission – Joint Research Center (JRC). A schematic is presented in Fig. 1 and details can be found in Zardini et al. (2016) and references therein. Here briefly, vehicles performed emission tests on a 48″ roller bench (Zoellner GmbH) by following prescribed driving cycles as described in the respective European Regulations 168/2013 and 134/2014 (EU, 2013a, 2014) and presented in section 2.3. PM and SPN23 emission measurements were performed based on these regulations and the so-called Particle Measurement Programme protocol (PMP, Andersson et al., 2007). The raw exhaust was diluted in a Constant Volume Sampling (CVS) tunnel (flow rate ≈ 5.5 m^3^/min) and a constant fraction of the diluted exhaust gas was sampled through 47 mm PTFE-coated glass-fiber filters (one for each driving cycle phase, average flow = 50 L/min – approximately 80 cm/s, filter temperature around 25 °C) in order to determine the deposited PM by gravimetric analysis. The length of the stainless-steel tube from the tailpipe to the dilution tunnel was 4–5 m (inner diameter d_in_ = 10 cm), leading to residence times of 5–15 s depending on flowrate. The setup and parameters fulfilled Regulation (EU) 134/2014 (2014).

The influence of the dilution tunnel parameters (e.g. dilution ratio, filter flow velocity) on PM mass determination has been thoroughly discussed in the literature (Vouitsis et al., 2003; Maricq et al., 2018). Based on previous studies we expect a decrease of the particle concentration and increase of the particle size due to agglomeration (Czerwinski et al., 2013). The decrease of particle concentration should be around 40% for diesel vehicles with high emissions (Isella et al., 2008) and 10–20% for motorcycles with emission levels close to the passenger cars limit (6 × 10^11^ km^−1^) (Giechaskiel et al., 2019b). High exhaust gas temperatures can also force deposited material to desorb from the tube walls and form new particles of volatile (Maricq et al., 1999) or semi-volatile character (Giechaskiel, 2019a, 2019b).

SPN23 emissions were determined using a PMP-compliant system (AVL Particle Counter - APC 489), sampling from the CVS (Giechaskiel et al., 2010). An additional Condensation Particle Counter (TSI CPC 3792) with a size cut-off at 10 nm was sampling in parallel (SPN10). SPN10 emissions in this case were corrected based on the PMP Particle Concentration Reduction Factor (PCRF) method. Finally, total particle number (TPN) emissions of all particles larger than 10 nm (TPN10), without distinction of particle volatility were determined either by an Engine Exhaust Particle Sizer (TSI EEPS 3090) sampling from the CVS or, when TPN concentrations were below the EEPS detection limit, by a TSI CPC3010 with an additional dilution stage (dilution ratio = 800 in most cases). In both cases no corrections for particle losses in sampling line were applied for the calculation of emission levels. In a few cases (some tests with vehicles 3, 4, 14, 25, 26 in Table 3) a heated catalytic stripper (CS) and an extra dilution stage were installed upstream the EEPS for volatile particles elimination and determination only of the solid particle number size distribution.

**Table 3:** Technical specifications of the test vehicles. Notes: 2S = 2-stroke, 4S = 4-stroke, G = gasoline, D = diesel, C = carburettor, PFI = port fuel injection, DI = direct injection, PCI = pre-chamber injection, 2WC = two-way oxidation catalyst, 3WC = three-way catalyst, DOC: diesel oxidation catalyst.

Vehicle type	Sub-category	Vehicle ID	Engine Type	Displ. [cm^3^]	Power [Kw]	Trans-mission	After-reatment	Reg. Year	Mileage [km]	Euro	Driving Cycles
2S Mopeds	L1e-A	1	2S-G-C	30	0.5	Fixed	none	2009	200	1	R47 WMTC 1
	L1e-B	2	2S-G-C	50	2	Manual	2WC	2015	120	2	R47 WMTC 1
	L1e-B	3	2S-G-C	50	3	CVT	2WC	2015	200	2	R47 WMTC 1
	L1e-B	4	2S-G-C	50	3	CVT	2WC	2015	500	2	R47 WMTC 1
	L1e-B	5	2S-G-C	50	3.2	Fixed	2WC	2010	200	2	R47 WMTC 1
	L1e-B	6	2S-G-C	50	3.3	CVT	2WC	2015	500	2	R47 WMTC 1
	L2e-U	7	2S-G-C	50	1.8	Manual	2WC	2016	100	2	R47 WMTC 1
4S Mopeds	L1e-B	8	4S-G-C	50	1.6	CVT	2WC	2012–2015	5500–6328	2	WMTC 1
	L1e-B	9	4S-G-C	49	2.4	CVT	2WC	2008–2015	614–8567	2	WMTC 1
	L1e-B	10	4S-G-C	50	2.5	CVT	2WC	2013	846	2	R47 WMTC 1
	L1e-B	11	4S-G-C	50	2.6	CVT	2WC	2013	4926	2	R47 WMTC 1
	L1e-B	12	4S-G-C	50	2.8	CVT	2WC	2010	300	2	R47 WMTC 1
	L1e-B	13	4S-G-C	50	2.9	CVT	2WC	2007–2015	1905–88040	2	WMTC 1
	L1e-B	14	4S-G-C	50	3	CVT	2WC	2015	200	2	R47 WMTC 1
Motorcycles	L3e-A1	15	4S-G-C	125	7	CVT	2WC	2012	1372	3	WMTC 1
	L5e-A	16	4S-G-C	197	7.5	Manual	2WC	2016	100	3	R40 WMTC 2-1
	L3e-A2	17	4S-G-PFI	155	9.5	CVT	3WC	2015	950	3	WMTC 2-1
	L3e-A1	18	4S-G-PFI	125	10.8	CVT	3WC	2010	200	3	WMTC 2-1
	L3e-A2	19	4S-G-PFI	300	16.3	CVT	3WC	2015	500	3	WMTC 2-2
	L3e-A2	20	4S-G-PFI	280	18.5	CVT	3WC	2015	2871	4	WMTC 2-2
	L3e-A2	21	4S-G-PFI	330	25	CVT	3WC	2012–2013	4657–10516	3	WMTC 3-1
	L3e-A2	22	4S-G-PFI	690	32	Manual	3WC	2016	1000	4	WMTC 3-2
	L3e-A3	23	4S-G-PFI	690	55	Manual	3WC	2016	13814-24940	3	WMTC 3-2
	L3e-A3	24	4S-G-PFI	1170	92	Manual	3WC	2015	1156	4	WMTC 3-2
Quads	L7e-B1	25	4S-G-PFI	570	11	CVT	2WC	2015	900	2	R40 WMTC 2-1
	L7e-B2	26	4S-G-PFI	700	15	CVT	2WC	2016	638	2	R40 WMTC 2-1
	L7e-B1	27	4S-G-PFI	980	15	CVT	3WC	2016	538	2	R40 WMTC 2-1
	L7e-B1	28	4S-G-PFI	450	16.9	CVT	3WC	2016	17	2	R40 WMTC 2-1
Minicars	L6e-BU	29	D-4S-DI	400	4	CVT	DOC	2015	988	2	R47 WMTC 2-1
	L6e-BP	30	D-4S-PCI	480	4	CVT	2WC	2015	120	2	R47 WMTC 2-1

### 2.2 Test vehicles

A sample of 30 L-category vehicles including 2-wheelers (mopeds and motorcycles), 3-wheelers (tricycles) and 4-wheelers (quads and minicars) was tested. These vehicles were chosen based on the market data analysis performed by Clairotte et al. (2016) in order to be as representative as possible of the EU circulating fleet, although high heterogeneity is observed among the different countries. For instance, the rate of mopeds and motorcycles per 1000 inhabitants in Greece is 275, while the respective number in France is only 65 (EU, 2018c). Significant differences are also observed in new vehicles registrations ratio between mopeds and motorcycles. In the period 2010–2018, the average ratio of motorcycles/mopeds registrations was 0.2 in Netherlands, while in Spain this ratio was 7.6 (ACEM, 2019a). Detailed technical specifications of the test vehicles are given in Table 3, while the specific characteristics and example vehicles for each sub-category are presented in Table 1 and Table S.1 (Supplementary Material), according to the terminology of Regulation 168/2013 (EU, 2013a). Mopeds were split into 2 groups according to the engine stroke, 2S and 4S, given their different behavior in terms of emissions (e.g., Zardini et al., 2014). 2S moped sub-category also contains a Euro 1 powered cycle (L1e-A) as it is equipped with 2S engine and belongs to L1e-A category. All the studied mopeds complied with Euro 2 emission standard, which was superseded by the Euro 3 standard in 2014. Taking into consideration that the estimated average L-category fleet age (vehicles in use for the period 2010–2040) is 8 years (Ntziachristos et al., 2017), Euro 2 mopeds are still considered the dominant environmental category in the EU circulating fleet (EU, 2013b; Ntziachristos et al., 2017). The same applies to quads and minicars. The motorcycles group included seven Euro 3 and three Euro 4 vehicles. The Euro 4 standard was introduced with Regulation EU 168/2013 (EU, 2013a) and was enforced in 2016, thus the share of Euro 3 motorcycles in current fleet is still high. A tricycle (sub-category L5e-A) was also included in the motorcycles sample, due to similar powertrain specifications. In the cases of vehicles 8, 9, 13, 21 and 23, three different vehicles of the same model were tested. For those vehicles, the average grouped emissions were assessed. All test vehicles were fuelled with certified E5 petrol or B7 diesel (EU, 2014).

### 2.3 Driving cycles

**Fig. 2:**
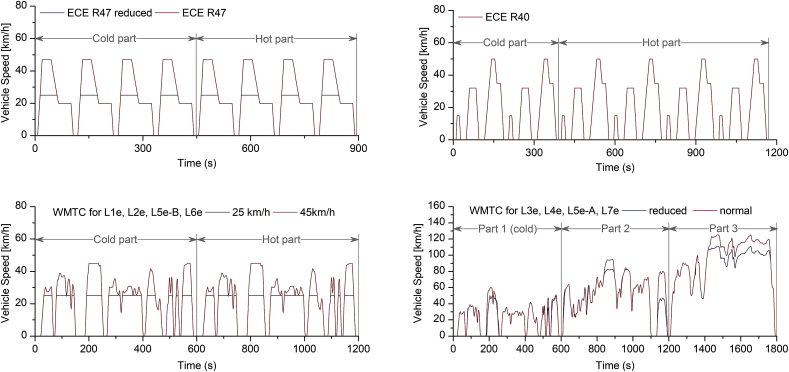
Driving cycles used in this study based on Regulation EU 134/2014 (EU, 2014). ECE R47 and R40 driving cycles are presented in upper panel. WMTC for sub-categories L1e, L2e, L5e-B and L6e and L3e, L4e, L5e-A, L7e are presented in lower panel. Different versions according to vehicle max speed are illustrated in each case.

The applicable driving cycles on L-category vehicles are summarized in Fig. 2 and described in Regulation EU 134/2014 (EU, 2014). The ECE R47 (for mopeds and minicars) and the ECE R40 (all the others) are the mandatory type approval driving cycles in EU up to the Euro 4 standard. Motorcycles up to Euro 3 could be type-approved with the R40 driving cycle, while Euro 4 motorcycles are type-approved with the worldwide-harmonized motorcycle test cycle (WMTC, optional for Euro 3 motorcycles). With the introduction of the Euro 5 package in 2020, all L-category vehicles will follow the WMTC which covers a wider engine map area compared to the R40 and R47 driving cycles (Ntziachristos et al., 2017). It should, however, be mentioned that even WMTC may not be representative of the real-world driving conditions, especially for some specific L-subcategories, such as the heavy all-terrain quads and the quadri-mobiles for utility purposes. The authors’ recommendation is that real-world driving emissions of these vehicle types should be monitored in the years to come, so that a new measurement methodology can be developed for those. On-road tests that were performed in the context of the Euro 5 effect study (Ntziachristos et al., 2017) with a Portable Emissions Measurement System (PEMS) revealed that on-road routes can include more comprehensive driving conditions and cover a wider engine operation area than WMTC, thus gaseous emissions may also be higher. Due to the complexity of particle measurement equipment as presented in section 2.1, technical and safety issues (e.g. size, need for high dilution and heated lines), these tests comprised only gaseous emissions measurements. For these reasons, no on-road particulate emission measurements are presented in the current study. A sensor-based system could solve these technical and safety issues, but such a system does not comply with the PEMS specifications prescribed by current regulation. In our study, we tested most vehicles over both the United Nations driving cycles (ECE R47 or R40) and the prescribed WMTC out of the 5 available classes determined by the vehicle category and its maximum declared speed as presented in Table S.2 in Supplementary Material. All WMTC versions, as defined for the different L-category vehicle classes, include a first phase of 600 s with vehicle starting from ambient conditions (cold phase, hereafter) and a second phase which is run without interruption in hot engine conditions. A third phase is run for vehicles with declared speed above 130 km/h. From the Euro 3 standard on, cold start emissions (those produced during the cold phase) are included in the evaluation of the emission factors to compare with limit values. However, we always sampled and analysed the cold phase in our study even for pre-Euro 3 vehicles in order to give more realistic emission factors. The calculation of driving cycle-average particulate emissions was performed based on the weighting factors presented in Table S.3 in Supplementary Material for the WLTC and ECE, according to the original prescriptions of Regulation EU 134/2014 (EU, 2014). Vehicles with manual transmission followed the gear shift prescriptions contained in the United Nations WMTC gearshift tool of the same regulation (EU, 2014).

## 3 Results and discussion

### 3.1 PM and SPN23 emissions, and size distributions

**Fig. 3:**
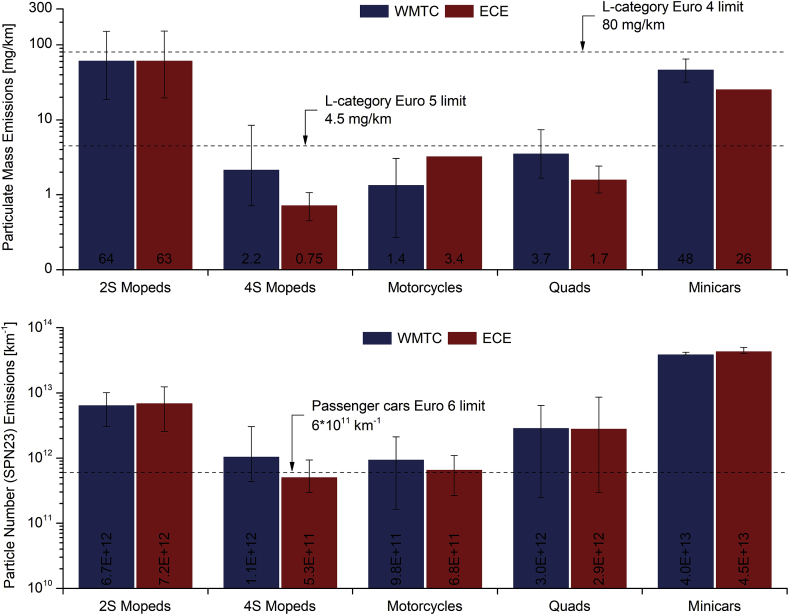
Particulate mass (PM, upper panel) and solid particle number with a size cut-off at 23 nm (SPN23, lower panel) emissions of each sub-category over the WMTC and ECE driving cycles. Euro 4 and 5 L-category PM limits (applicable to CI and GDI engines only) and Euro 6 passenger cars SPN23 legislation limits are included (dashed lines). Error bars refer to min-max emission values among the vehicles within each sub-category.

Fig. 3 presents the average PM and SPN23 emissions for each sub-category over cold-start Euro 5 WMTC and Euro 4 ECE driving cycles (and associated weighting factors described in section 2.3), with error bars corresponding to minimum and maximum emission levels among the vehicles. L-category and passenger cars legislation limits are also presented. The variation among the different test repetitions is represented by the coefficient of variation (CV, as average of all vehicles within each sub-category) in Figure S.1 in Supplementary Material. In most cases CV was below 25% except from the case of WLTC tests with quads, where CV values above 45% are observed. CV levels were quite similar between PM and SPN23 emissions, while in most cases higher CV was observed in WMTC compared to ECE.

Two-stroke mopeds and diesel minicars comprised the vehicles with the highest particulate emissions, exceeding the Euro 5 PM limit (4.5 mg/km, applicable to diesel and DI engines) by 14 and 11 times, respectively, over the WMTC. The corresponding SPN23 emissions were found to be 11 and 67 times higher, respectively, than the Euro 6 limit of passenger cars (6 × 10^11^ km^−1^). The studied minicars were equipped with non-DPF diesel engines; hence they were unsurprisingly among the highest emission vehicles. High particulate emissions from 2S mopeds are due to cylinder scavenge losses (Oswald and Kirchberger, 2018) and are in agreement with other studies. Ntziachristos et al. (2003) and Martini et al. (2009) reported PM emission levels above 100 mg/km for pre-Euro 2S moped, while Euro 2 carburettor 2S mopeds produced up to 50 mg/km of PM over the cold-start ECE R47 driving cycle (Martini et al., 2009; Adam et al., 2010). Favre et al. (2011) found that PM emissions from a Euro 3 2S moped were more than 12 mg/km and SPN23 emissions levels were close to 10^14^ km^−1^ during WMTC. Finally, Giechaskiel et al. (2015) exhibited SPN23 emissions from Euro 2 2S mopeds close to 10^13^ km^−1^ under the cold part of ECE R47.

Focusing on PM emissions from the other sub-categories, these were in all cases one order of magnitude lower than 2S mopeds and diesel minicars, complying with the L-category Euro 5 limit (4.5 mg/km), even if this is not applicable to these categories. The large difference in PM emissions (up to one order of magnitude) between 2S and 4S engines was also shown by the studies of Martini et al. (2009) and Favre et al. (2011), who evaluated several Euro 1-3 L-category vehicles. PM emissions of the studied motorcycles are in agreement to the study of Costagliola et al. (2016), who found that PM emissions of a Euro 3 motorcycle were approximately 1.5 mg/km. In addition, Favre et al. (2009) evaluated 4 Euro 3 motorcycles and exhibited emissions in the range of 0.6–2.2 mg/km.

With regard to SPN23 emissions, 4S mopeds, motorcycles and quads were lower than 2S mopeds and minicars, but still above the 6 × 10^11^ km^−1^ limit in most cases. Emission levels of 4S mopeds over WMTC (11 × 10^11^ km^−1^) are consistent to the study performed by Favre et al. (2011) on Euro 3 vehicles. Giechaskiel et al. (2015) exhibited SPN23 emissions from 4S mopeds above 20 × 10^11^ km^−1^ during the cold part of ECE and close to 6 × 10^11^ km^−1^ limit (except for one vehicle) over the hot part. SPN23 emissions from motorcycles of the current study were found to be 1.1–1.6 times higher than the limit and this is consistent to the study of Favre et al. (2009) who found SPN23 emissions in the range 2–9 × 10^11^ km^−1^ over the WMTC. Ntziachristos and Galassi (2014) reported even higher SPN emission factors for motorcycles (12 × 10^11^ km^−1^) based on a review of relevant studies. The studied quads exceeded the Euro 6 passenger cars limit by 5 times, an observation that is in agreement with the studies performed by Giechaskiel et al. (2015) and Ntziachristos et al. (2008).

Particulate emissions did not significantly vary between the two driving cycles in most cases of SPN23 emissions, while higher differences were detected in PM. In 4S mopeds, quads and minicars, PM emissions over the WMTC were (1.8–3 times) higher than with the ECE driving cycles, but the opposite trend was observed in motorcycles (PM data over ECE available only for 1 motorcycle), while with 2S mopeds, WMTC and ECE driving cycles were at the same level. Notably, when a sub-category complied with the PM or SPN23 limit on the WMTC, it also complied with the ECE driving cycle, except for the case of SPN23 of 4S mopeds. This trend was confirmed, when emissions of specific vehicles were separately taken into account.

**Fig. 4:**
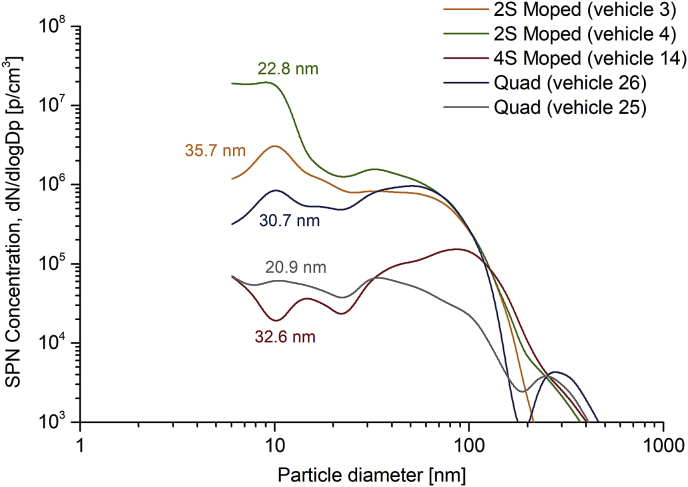
Solid particle number size distribution over the WMTC for 5 L-category vehicles (two 2S mopeds, one 4S moped and two quads). Values on each curve correspond to geometric mean diameter.

Fig. 4 presents the solid particle number size distribution of 5 L-category vehicles (2S and 4S mopeds and quads) over the WMTC with related geometric mean diameter (GMD), which varies among the different vehicles in the range between 20.9 and 35.7 nm, while variations are also observed among vehicles of the same sub-category. The studies of Giechaskiel et al. (2015) and Czerwinski et al. (2010) on 2S and 4S mopeds reported similar GMD levels, in the range of 20–40 nm. The rather low GMD values indicate that sub-23 nm area may comprise a significant contributor in SPN emissions, thus an SPN23 limit may omit a great part of emitted particles. This finding is further analysed in section 3.3.

### 3.2 Towards the Euro 5 step

**Fig. 5:**
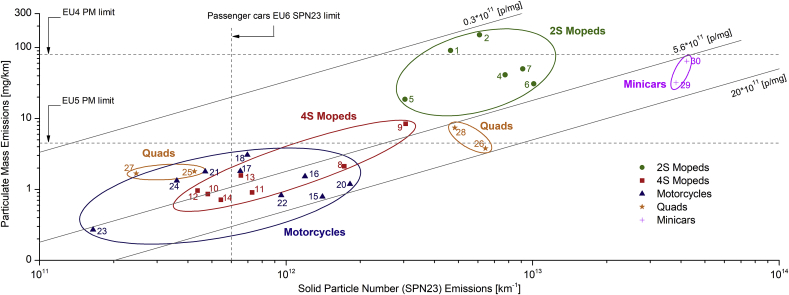
Particulate mass (PM) vs solid particle number with a size cut-off at 23 nm (SPN23) emissions of each studied vehicle. Euro 4 and 5 L-category PM limits and Euro 6 passenger cars SPN23 limit are illustrated. Vehicles are grouped based on sub-categories presented in Table 3. Ellipses are built around category data points to guide the eyes.

The Euro 5 standard introduces a PM limit of 4.5 mg/km for all L-category vehicles equipped with CI or GDI engine. However, as previously shown, other vehicles also produced high PM (2S mopeds), while SPN23 emissions were in most sub-categories close or above the Euro 6 passenger cars limit (5 and 12 times higher in the case of quads and 2S mopeds respectively). Fig. 5 presents PM and SPN23 emission levels of each test vehicle (Euro 1–4) under cold-start WMTC. An overall linear fit of 5.6 × 10^11^ p/mg can be established with rather large inter-vehicle variations which envelop the data in the range between 0.3 × 10^11^ and 20 × 10^11^ p/mg. The best fit is lower than the experimental fits of Giechaskiel et al. (2012) review (mainly diesel light-duty and heavy-duty vehicles), Joshi and Johnson (2018) (mainly GDIs) of around 20 × 10^11^ p/mg, and Giechaskiel et al. (2019c) of 12 × 10^11^ p/mg (Euro 4 motorcycles). Thus, the lower fit for L-category vehicles means either a larger mean size of particles (unlikely) or a higher fraction of volatile material in the PM mass (most likely), than in previous cases.

The following discussion assesses whether the Euro 5 PM limit should be extended to other engine technologies and if the introduction of a SPN limit, in analogy to light duty vehicles, should be applied to some L-sub-categories.

#### 3.2.1 2-Stroke mopeds

Two-Stroke mopeds (sub-category L1e-B, including L1e-A and L2e-U vehicles) had the highest PM (14 times higher than 4.5 mg/km limit in both WMTC and ECE) and the second highest SPN23 emissions of the current study (11 and 12 times higher than Euro 6 passenger cars limit in WMTC and ECE, respectively). All the studied 2S mopeds (Euro 1 and 2) were equipped with carburetted fuel system, which is the dominant technology in the mopeds circulating fleet, and so the Euro 5 PM limit would not be applicable to them. Nevertheless, it is expected that 2S mopeds market share will be significantly reduced in Euro 5 step (4S with fuel injection are expected instead) due to their inability to comply with total hydrocarbon (THC) emission limits (Ntziachristos et al., 2017). Particulate and HC emissions are closely correlated as showed by previous studies (Martini et al., 2009; Spezzano et al., 2009; Ntziachristos et al., 2017) and confirmed by the results presented in Figure S.2 in Supplementary Material, which presents the THC-PM correlation for 2-S mopeds over the WMTC and ECE driving cycles. Thus, it is expected that the reduction of THC emissions in Euro 5 step will also have a positive impact on particulate emissions. However, it is important that if 2S will be type-approved under Euro 5, these will continue to be monitored in the following years in order to confirm the expectations. Emissions reductions can be achieved with advanced fuel injection systems, such as electronic carburettor or direct injection systems with secondary air injection HC control (Ntziachristos and Galassi, 2014; Winkler et al., 2016), optimized lube oil dosing and high-quality oil (e.g. synthetic oil with low Sulphur and PAH content) and improved fuel (e.g. alkylate fuel or gasoline-ethanol blends) (Ålander et al., 2005; Czerwinski et al., 2009; Morin et al., 2011; Zardini et al., 2014).

#### 3.2.2 4-Stroke mopeds

As regards 4S mopeds (sub-category L1e-B, all Euro 2 compliant), PM emissions would already comply with the Euro 5 limit in all cases except for vehicle 9, although this limit would not be applicable because none of the vehicles is equipped with CI or GDI engine. Vehicle 9 also exceeded (by 5 times) the Euro 6 passenger cars SPN23 emission limit, while vehicle 8 exceeded this limit by 3 times. Interestingly, PM emissions of this vehicle were very close to the 4.5 mg/km limit value, indicating that even if the Euro 5 PM limit would be extended, it might not be enough to exclude vehicles with high SPN23 emissions. Similarly to the case of 2S mopeds, a reduction of 4S mopeds market share is expected in Euro 5 step, especially due to the stricter THC limit compared to Euro 4 (Ntziachristos et al., 2017). THC and particulate emissions can be reduced through improved fuel injection with shorter injection periods to avoid wall and piston wetting and high quality fuel, such as alkylate gasoline (Zardini et al., 2014) and ethanol blends (Czerwinski et al., 2010), but this is expected to increase cost that would further decrease their already dropping market size.

#### 3.2.3 Motorcycles

In the case of motorcycles (sub-categories L3e-A1, L3e-A2 and L3e-A3) PM emissions were 1.5–17 times lower than the forthcoming Euro 5 limit for all studied vehicles (Euro 3 and 4), in the range 0.27–3 mg/km. Note that this limit will not be applicable to the test vehicles as none was equipped with CI or GDI engine.

Large differences in SPN23 emissions were detected among the studied motorcycles, varying between 2 × 10^11^ km^−1^and 20 × 10^11^ km^−1^. Focusing on Euro 4 (vehicles 20, 22 and 24) two out of three vehicles exceeded the 6 × 10^11^ km^−1^ limit (50% and 3 times higher), although, as already mentioned, PM emissions were well below the 4.5 mg/km limit. This indicates that even if the Euro 5 PM limit was extended to cover PFI motorcycles, vehicles with high SPN emissions might still not be identified. It should, however, be noted that similarly to the case of mopeds, THC emissions from motorcycles are expected to decrease in Euro 5 step in order to comply with the stricter limits (74% reduction compared to Euro 4, for vehicles with maximum speed lower than 130 km/h), thus it could be expected that particulate emissions will also decrease.

#### 3.2.4 Quads

As presented in Fig. 5, two separate emission groups were formed within this sub-category (L7e-B), due to different SPN23 emissions. In the first group (vehicles 25 and 27), SPN23 emissions were well below the Euro 6 passenger cars limit (in the range 2.5–4.3 × 10^11^ km^−1^), while in the second (vehicles 26 and 28) these were one order of magnitude higher. This high difference can be attributed to the different application type of each vehicle. High emissions of vehicle 26 (sport, side-by-side buggy, designed for high-velocity trail passing) for instance, are attributed to fuel enrichment, as revealed by the HC emissions being 3 times higher than the average of the other quads. On the other hand, the low-pollutant vehicles (25 and 27) were all-terrain vehicles (ATVs), with higher center of gravity and less sporty character.

PM emissions were found to be in a relatively narrow range (2–7 mg/km), even for the vehicles with high SPN23 emissions. Euro 5 PM limit (4.5 mg/km) will not be applicable to any of these vehicles because they were all equipped with PFI gasoline engines (Euro 2). The discrepancy between PM and SPN23 emissions reveals that, even if the PM limit is to be applied on PFI quads, vehicles with high SPN emissions will not be identified. Despite their low market share (EU, 2010), quads are used in tourist areas, mountains and seashore paths which supposedly offer high air quality standards. Thus, special attention should be focused on those in order to identify any potentially high emission vehicles. Towards this direction, the introduction of a SPN limit for this sub-category should be investigated.

#### 3.2.5 Minicars

SPN23 emissions of diesel minicars (sub-category L6e-B, Euro 2) were 2 orders of magnitude higher than Euro 6 passenger cars limit, while PM emissions were also high, in the range of 32–65 mg/km. They complied with the lenient Euro 4 PM limit, but as they fall in the scope of the Euro 5 PM limit of 4.5 mg/km, manufacturers will have to significantly improve powertrain technologies and probably install DPF, in analogy to the measures applied on light-duty vehicles (Ntziachristos and Galassi, 2014; Oso et al., 2017). These measures will increase cost and complexity, while space limitations should be taken into consideration (Ntziachristos et al., 2017). As a consequence of particularly demanding technical requirements, this subcategory assumed derogation from Euro 5, which was postponed to 2022 (EU, 2018b, 2018a). Another possible scenario is the electrification of minicars either in terms of fully or hybrid electric powertrains (Cahill, 2013; Santucci et al., 2016; Ntziachristos et al., 2017). If hybrids will be equipped with CI or GDI engines, optimization to comply with the Euro 5 limit will still be required.

Although minicars currently represent a mere 1% of the L-category fleet and therefore their impact on emissions is low in absolute values (EU, 2010; Ntziachristos et al., 2017), close attention is needed as these vehicles are mainly driven in or close to the city center. In addition, minicars are expected to become a popular means of transport in modern dense cities (Cahill, 2013; Pavlovic, 2015; Karaca et al., 2018), due to their small size, low fuel consumption, comfort comparable to small passenger cars, and reduced administrative burden (they can be driven from the age of sixteen in most European countries). The quantification of the potential impact of these vehicles on air pollution in cities would depend on the size and composition of the urban fleet. However, even if the impact would be small, the policy-related question remains on whether the authority allows the circulation of a private 2-seater which pollutes like old non-DPF diesel vehicles. Finally, minivans, which belong to the same category, are expected to be widely used as a means of commercial transportation in big cities (Cahill, 2013), while municipal activities (e.g. street cleaning and waste collection) can also be served by this vehicle type.

#### 3.2.6 Final remarks

Summarizing the above-presented analysis, the authors’ opinion is that the Euro 5 PM limit should be extended to vehicles with 2S engines or a SPN limit should be introduced, as their emission levels were found to be close to diesel vehicles. In the case of 4S mopeds, motorcycles and quads, PM emissions are already at low levels, but this is not the case for SPN23 emissions. Taking into account that the limit of 4.5 mg/km corresponds to at least 60 × 10^11^ km^−1^ (based on Fig. 5, Giechaskiel et al., 2012, 2019c and Joshi and Johnson, 2018) i.e. >10 times above the Euro 6 passenger cars SPN23 limit, a PM limit will not be sufficient to detect vehicles with SPN23 emissions above the current level for passenger cars.

The increase in the sales of L-category vehicles (mainly due to motorcycles) (Dorocki, 2018; ACEM, 2019b) and the reduction of passenger cars SPN23 emissions due to stricter legislation (Williams and Minjares, 2016), are expected to increase the relative contribution of L-category to vehicular SPN emissions during the next years. Thus, L-category vehicles will represent a major polluter in terms of SPN, especially in urban areas. For this reason, it is important that current Euro 4 and Euro 5 vehicles should come under scrutiny in order to assess whether further actions, such as the introduction of a SPN limit will be required. The same applies on diesel minicars, as their SPN emissions will not be subject to any regulation limit either.

### 3.3 Sub-23 nm and total particles

**Fig. 6:**
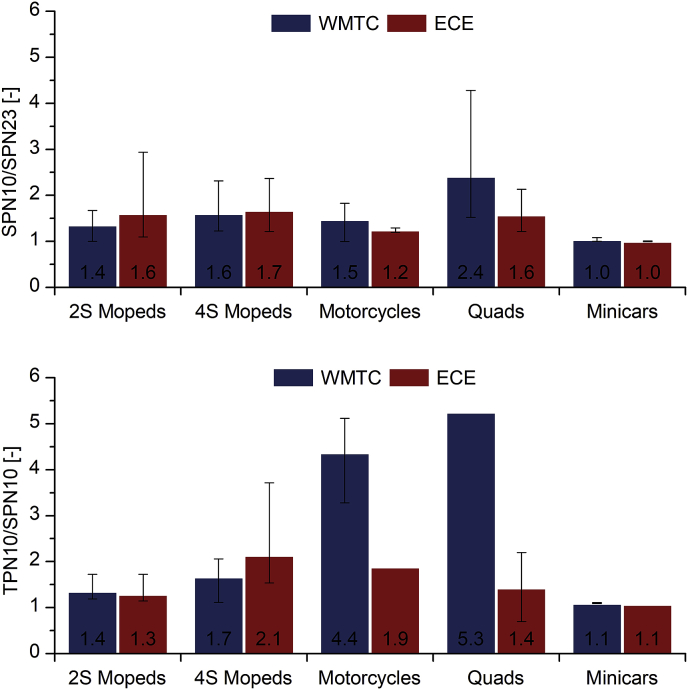
Solid particle number (SPN) emissions with a size cut-off at 10 nm (SPN10) over SPN emissions above 23 nm (SPN23) (upper graph) and total (solid and volatile) particle number emissions with a size cut-off at 10 nm (TPN10) over SPN10 (lower graph) for each sub-category over the WMTC and ECE driving cycles. Error bars refer to min-max ratios observed among the vehicles within each sub-category.

Fig. 6 presents SPN10/SPN23 (upper graph) and TPN10/SPN10 (lower graph) emissions ratios for each sub-category over the WMTC and ECE driving cycles. A high range for both ratios can be observed among the different sub-categories. Focusing on SPN10 emissions, these were almost identical to SPN23 emissions of diesel minicars and this is in agreement with the low sub-23 nm SPN fraction observed in diesel passenger cars without DPF (Giechaskiel et al., 2017, 2018). On the other hand, a high SPN10/SPN23 ratio (in the order of 2.4) is observed in quads over the WMTC. In the other sub-categories, SPN10 emissions were 25–67% higher than SPN23 emissions. Similar findings were exhibited by Giechaskiel et al. (2015) for L-category vehicles, who found that particles in the size range 10–23 nm comprise 10–80% of the emissions above 23 nm, although significantly higher fractions can be observed in some vehicles. These percentages have not been corrected for size-specific particle losses below 30 nm, which could almost double the reported percentages (Giechaskiel et al., 2019a, 2019b, 2019c, 2019d). TPN10/SPN10 ratio significantly varies among the different sub-categories, especially over the WMTC. The highest ratios are observed in quads and motorcycles, with TPN10 emissions being 5.3 and 4.4 times higher than SPN10 respectively. On the other hand, in diesel minicars TPN10/SPN10 ratio is close to 1, indicating that diesel exhaust is dominated by solid particles above 23 nm.

**Fig. 7:**
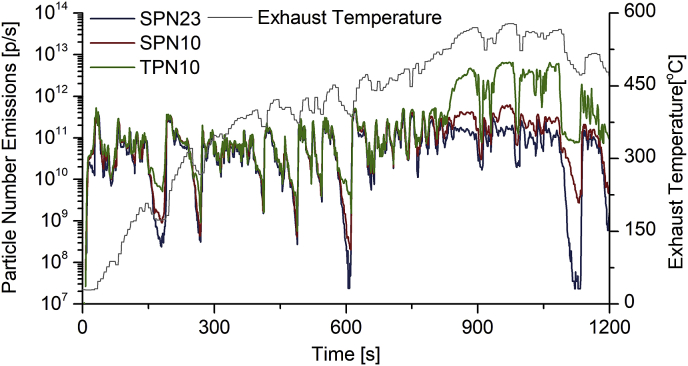
Time series of SPN with a cut-off size at 10 and 23 nm (SPN10 and SPN23 respectively) and total (solid and volatile) particle number with a cut-off at 10 nm (TPN10) over cold-start WMTC are presented. Exhaust temperature at vehicle tailpipe is also illustrated.

These findings indicate that sub-23 nm area may comprise a significant part of particulate emissions from L-category vehicles, either as solid or volatile particles. Nevertheless, measurement of solid and volatile particles in this area may be prone to artifacts, which can occur through release of material from the transfer line walls during high exhaust temperature events, especially from vehicles that generally emit high quantities of such components (Maricq et al., 1999; Ntziachristos et al., 2004; Giechaskiel, 2019a). The artifact formation potential may be visualized by looking at the emissions time series of a quad vehicle (vehicle 26, cold-start WMTC) presented in Fig. 7. A clear increase of SPN10 and especially TPN10 emissions can be observed during the last part of the driving cycle (800–1200s) compared to SPN23 emissions, when exhaust temperature is at its highest (up to 580 °C). This may be the reason for the high SPN10 and TPN10 fractions in quads presented in Fig. 6.

Whatever the source of volatile and semi-volatile fraction is, the current SPN23 measurement methodology prescribed by PMP (Andersson et al., 2007) may not be sufficient in eliminating high fractions of these species. Consequently, artifacts in solid particle emissions measurements may also occur through nucleation of these volatile and semi-volatile species, and this is expected to be of importance in sub-23 nm area (Giechaskiel et al., 2015). Thus, for L-category vehicles a catalytic stripper is recommended for sub-23 nm measurements. Recent studies also suggested that using a mixing tee or a transfer tube in open configuration can minimize these artifacts due to the reduction of exhaust gas temperature (Giechaskiel, 2019b; Giechaskiel et al., 2019d).

### 3.4 Cold start effect

**Fig. 8:**
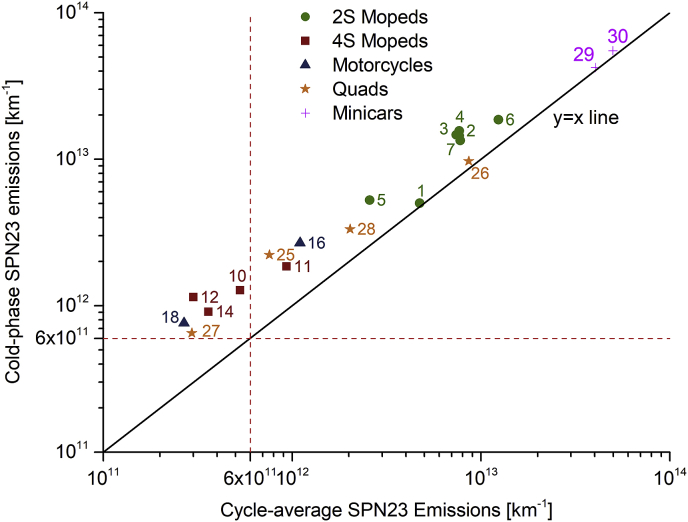
Solid particle number with a size cut-off at 23 nm (SPN23) emissions over the cold phase (vertical axis) and the whole ECE (R40 or R47) driving cycle duration (horizontal axis).

Fig. 8 presents the absolute SPN23 emission levels over the cold phase (vertical axis) and during the whole ECE driving cycle (horizontal axis), chosen because its cold and hot parts have identical speed profiles. The Euro 6 passenger cars SPN23 limit and the y = x line are also illustrated. As expected, the relative cold effect is significantly higher in overall low emission vehicles compared to those with high emissions, in which cycle-average emissions were almost at the same level as in the cold phase: low emitters produce the large part of pollutants in the cold phase. An interesting observation is derived from vehicles 10, 12, 14, 18, 27, in which cycle-average emissions were below the passenger cars limit, but cold phase emissions were found to be by up to 2.2 times higher than this limit. Taking into consideration that several cold or semi-cold events may occur during the day under real-world operation (due to quick cooling of low-mass L-category engines), these findings indicate that operation under cold engine conditions may significantly contribute on L-category particulate emissions.

**Fig. 9:**
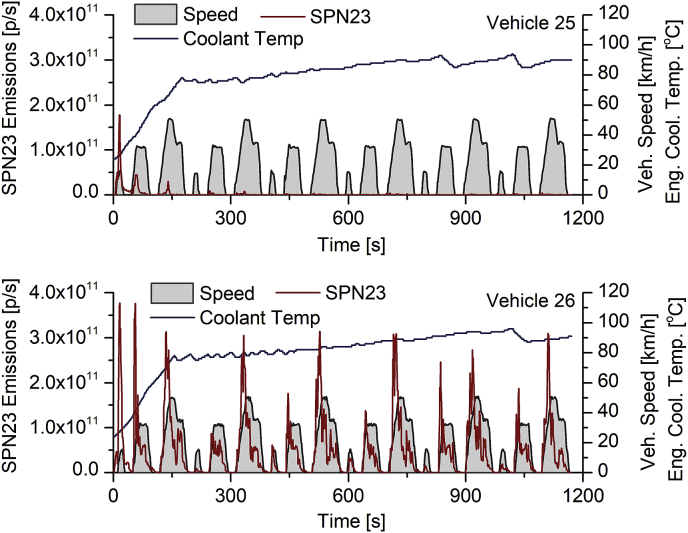
SPN23 emissions of two extreme cases (high and low cold effect in upper and lower graphs, respectively) within the quads sub-category. Vehicle speed and engine coolant temperature are also illustrated.

However, significant variations can be observed even within the same sub-category as shown in Fig. 9, which illustrates SPN23 emissions over the cold-start ECE R40 driving cycle for two quads. In the first vehicle (vehicle 25, upper graph) 87% of cumulative SPN23 emissions are ascribed to the cold phase (first two elementary modes of the ECE driving cycle). In the second vehicle (vehicle 26, lower graph), SPN23 emissions were consistently high during the whole cycle duration, with only 38% of cumulative SPN23 being emitted during the cold phase. This is attributed to fuel enrichment events during the whole driving cycle duration (THC emissions were 8 times higher compared to the first vehicle).

## 4 Conclusions

The current study presented particulate emissions measurements from 30 L-category vehicles complying with Euro 1–4 standards and being representative of the current circulating fleet in EU. The objective was to identify the highest polluters and assess whether further measures (e.g. extension of the PM limit to other engine types and/or the introduction of a SPN limit) are needed for the Euro 5 step in order to ensure that only clean vehicles penetrate in the market.

Emissions during prescribed driving cycles on a roller bench showed that 2S mopeds (sub-category L1e-B) and diesel minicars (sub-category L6e-B) comprised the highest PM and SPN23 emission vehicles. The Euro 5 PM limit (4.5 mg/km) will be applicable to diesel minicars, hence significant emissions reductions, through engine and after-treatment upgrades, are expected if such vehicles still exist in Euro 5 step. The extension of this limit (and or the introduction of a SPN23 limit) is proposed for 2S vehicles as well, in order to achieve emission reductions before they can enter the market. PM emissions of the other sub-categories and engine types were already below or close to the Euro 5, while additional engine and after-treatment upgrades are expected on Euro 4 and 5 steps.

An interesting finding was revealed by SPN23 emissions, which in most cases exceeded the Euro 6 passenger cars SPN23 limit. Along with 2S and diesel engines, SPN23 emissions of 4S PFI engines (e.g. quads, motorcycles) were up to 5 times higher than the Euro 6 passenger cars limit and this was observed even in the case of recent Euro 4 motorcycles. These findings indicate that a PM limit alone may not be sufficient to ensure the type approval of clean vehicles in the market and therefore the introduction of a SPN limit may be required. However, as this conclusion was based on Euro 4 vehicles, the recommendation should be confirmed by emissions of actual Euro 5 vehicles.

Sub-23 nm particles comprised a significant part of emitted particles, with average SPN10/SPN23 ratio being up to 2.4 for quads. Emissions of total (solid and volatile) particles also appeared to be high in the sub-23 nm area (TPN10/SPN10 = 4.4 and 5.3 from motorcycles and quads, respectively, over the WMTC). Nevertheless, special attention is needed with the interpretation of these results in order to avoid artifacts interference, especially due to the large volatile exhaust gas fraction. Finally, cold start was found to be a major contributor to SPN23 emissions, with cold phase emissions being up to 2.2 times higher than the passenger cars limit for the vehicles with cycle-average emissions below this limit.

## Disclaimer

The opinions expressed in this manuscript are those of the authors and should in no way be considered to represent an official opinion of the European Commission. Mention of trade names or commercial products does not constitute endorsement or recommendation by the authors of the European Commission.
