# Supplementary material for: Particulate emissions from L-Category vehicles towards Euro 5
Source: Environ Res. 2020 Mar;182:109071. doi: 10.1016/j.envres.2019.109071 (PMC7043005; doi:10.1016/j.envres.2019.109071)
Supplement: Multimedia component 1 [file mmc1.docx]

Supplementary Material for paper

**Particulate emissions from L-Category vehicles towards Euro 5**

Kontses^a^, A., Ntziachristos^a^, L., Zardini^b^, A. A., Papadopoulos^c^, G., Giechaskiel^b*^, B.

^a^ Laboratory of Applied Thermodynamics, Aristotle University of Thessaloniki,
P.O. Box 458, GR 54124, Thessaloniki, Greece, e-mails: [akontses@auth.gr](mailto:akontses@auth.gr) (Kontses A.), [leon@auth.gr](mailto:leon@auth.gr) (Ntziachristos L.)

^b^ European Commission Joint Research Center, Directorate for Energy, Transport and Climate, Sustainable Transport Unit, 21027 Ispra (VA), Italy, e-mails: [Barouch.GIECHASKIEL@ec.europa.eu](mailto:Barouch.GIECHASKIEL@ec.europa.eu) (Giechaskiel B.), [Alessandro.ZARDINI@ec.europa.eu](mailto:Alessandro.ZARDINI@ec.europa.eu) (Zardini A.Α.)

^c^ Emisia S.A., Antoni Tritsi 21, PO Box 8138, GR-57001, Thessaloniki, Greece, e-mail: [giorgos.p@emisia.com](mailto:giorgos.p@emisia.com) (Papadopoulos G.)

* Corresponding author. Tel.: +39 0332 78 5312, e-mail address: [Barouch.GIECHASKIEL@ec.europa.eu](mailto:Barouch.GIECHASKIEL@ec.europa.eu)

# Tables

Table S.1: L-category family details based on Regulation 168/2013 (EU, 2013). Sub-categories, key characteristics and example vehicles (no commercial scope, just examples) are provided in each case. Notes: PI: Positive Ignition, CI: Compression Ignition

| **Sub-category** | **Key characteristics** | **Example vehicles** |
| --- | --- | --- |
| **L1e-Le7, L-category vehicles** | | |
| All | - length ≤ 4 m (3 m for L6e-B, 3.7 m for L7e-C) - width ≤ 2 m (1m for L1e, 1.5 m for L6e-B, L7e-C) - height ≤ 2.5 m | 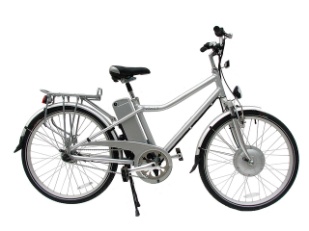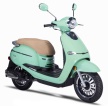 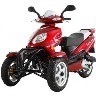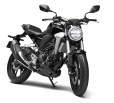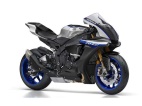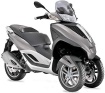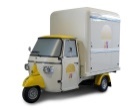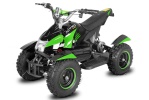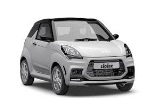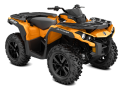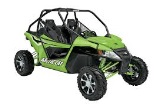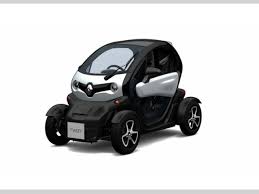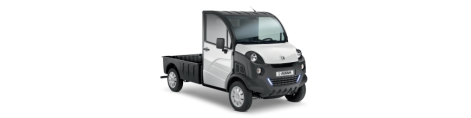 |
| **L1e, Light two-wheel powered vehicle** | | |
| L1e-A  Powered cycle | 2 wheels, ≤ 50 cc (PI), ≤ 25 km/h, ≤ 1 kW | 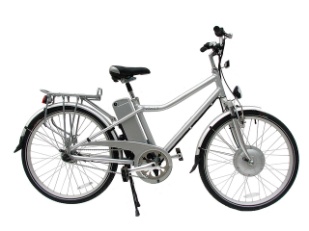 |
| L1e-B  Two-wheel moped | 2 wheels, ≤ 50 cc (PI), ≤ 45 km/h, ≤ 4 kW | 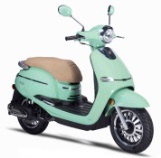 |
| **L2e, Three-wheel moped** | | |
| L2e-P  Three-wheel moped for passenger transport | 3 wheels, ≤ 50 cc (PI) or ≤ 500 cc (CI), ≤ 45 km/h, ≤ 4 kW, ≤ 270 kg, max 2 seating positions (incl. driver) | 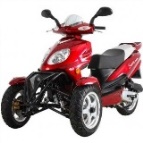 |
| L2e-U  Three-wheel moped for utility purposes | Same as L2e-P, exclusively designed for carriage of goods, loading bed area capable of carrying a min. volume represented by a 600 mm cube | 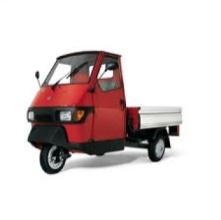 |
| **L3e, Motorcycle (two-wheel vehicle that cannot be classified as L1e)** | | |
| L3e-A1  Low-performance motorcycle | 2 wheels, ≤ 125 cc, ≤ 11 kW, ≤ 0.1 kW/kg | 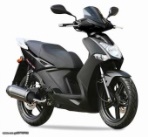 |
| L3e-A2  Medium-performance motorcycle | 2 wheels, ≤ 35 kW, ≤ 0.2 kW/kg | 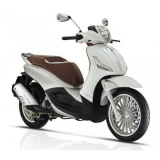 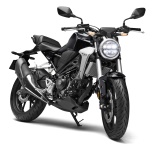 |
| L3e-A3  High-performance motorcycle | 2 wheels, > 35 kW, > 0.2 kW/kg | 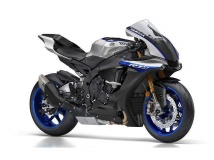 |
| L3e-AxE  Enduro Motorcycles | 2 wheels, seat height ≥ 900 mm, ground clearance ≥ 310 mm, ≤ 140 kg, no passenger | 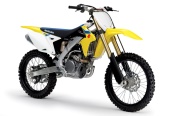 |
| L3e-AxT  Trial Motorcycles | 2 wheels, seat height ≥ 700 mm, ground clearance ≥ 280 mm, ≤ 100 kg, no passenger | 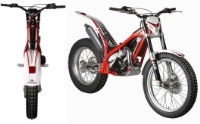 |
| **L4e, Two-wheel motorcycle with side-car** | | |
| No sub-categories | 3 wheels (asymmetric), characteristics equivalent to L3e | 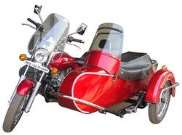 |
| **L5e, Powered tricycle (three-wheel vehicle that cannot be classified as L2e)** | | |
| L5e-A  Tricycle | 3 wheels (symmetric), ≤ 1000 kg, max 5 seats (incl. driver) | 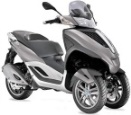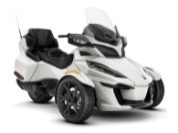  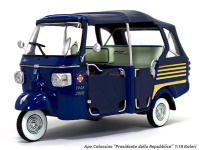 |
| L5e-B  Commercial tricycle | 3 wheels (symmetric), ≤ 1000 kg, max 2 seats (incl. driver), designed for utility purposes, loading bed area capable of carrying a min. volume represented by a 600 mm cube | 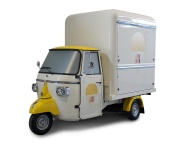 |
| **L6e, Light quadricycle** | | |
| L6e-A  Light on-road quad | 4 wheels, ≤ 45 km/h, ≤ 425 kg, ≤ 50 cc (PI), ≤ 500 cc (CI), max 2 seats (incl. driver), ≤ 4 kW | 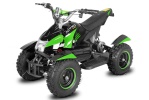 |
| L6e-B  Light quadri-mobile | 4 wheels, ≤ 45 km/h, ≤ 425 kg, ≤ 50 cc (PI), ≤ 500 cc (CI), max 2 seats (incl. driver), ≤ 6kW, enclosed driver and passenger compartment accessible by max 3 sides | L6e-BP  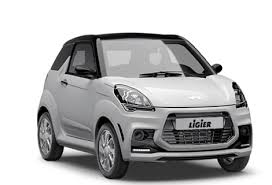  L6e-BU  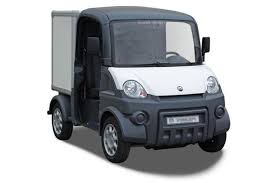 |
|  | Sub-sub-categories |  |
|  | L6e-BP: mainly designed for passengers  L6e-BU: exclusively designed for goods |  |

| **L7e, Heavy quadricycle (vehicle that cannot be classified as L6e)** | | |
| --- | --- | --- |
| L7e-A  Heavy on-road quad | 4 wheels, ≤ 450 kg, only transport of passengers, ≤ 15 kW | L7e-A1  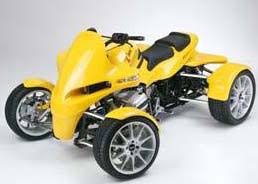  L7e-A2  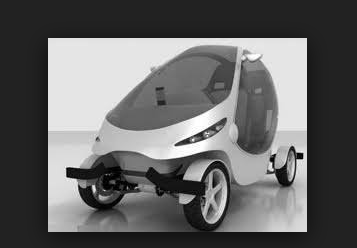 |
|  | Sub-sub-categories |  |
|  | L7e-A1: max 2 straddle seats (incl. driver), handlebar  L7e-A2: max 2 non-straddle seats (incl. driver) |  |
| L7e-B  Heavy all-terrain quad | 4 wheels, ≤ 450 kg (passengers), ≤ 600 kg (goods), ≤ 15 kW, ground clearance ≥ 180 mm | L7e-B1  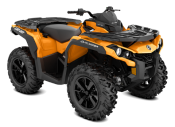  L7e-B2  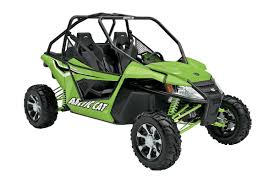 |
|  | Sub-sub-categories |  |
|  | L7e-B1 (all-terrain quad): max 2 straddle seats (incl. driver), handlebar, ≤ 90 km/h  L7e-B2 (side-by-side buggy): max 3 non-straddle seats (incl. driver), 2 of them side by side |  |
| L7e-C  Heavy quadri-mobile | 4 wheels, ≤ 450 kg (passengers), ≤ 600 kg (goods), ≤ 15 kW, ≤ 90 km/h, enclosed driver and passenger compartment accessible by max 3 sides | L7e-CP  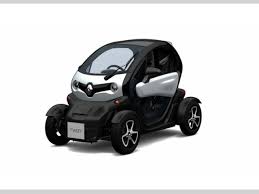  L7e-CU  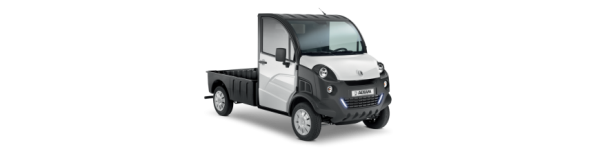 |
|  | Sub-sub-categories |  |
|  | L7e-CP (passenger transport): max 4 non-straddle seats (incl. driver),  L7e-CU (utility purposes): exclusively designed for goods, max 2 non-straddle seats (incl. driver) |  |

Table S. 2: Applicable WMTC Class based on vehicle class according to Regulation 134/2014 (EU, 2014).

| **Vehicle sub-category** | **WMTC Class** | **WMTC Parts** | **Applicable on vehicles** |
| --- | --- | --- | --- |
| L1e, L2e, L5e-B, L6e | 1 | Cold Part + Hot Part | 1-14, 29,30 |
| L3e, L4e, L5e-A, L7e | 1 | Cold Part 1 reduced speed + Hot Part 1 reduced speed | 15 |
|  | 2-1 | Cold Part 1 reduced speed + Hot Part 2 reduced speed | 16-18, 25-28 |
|  | 2-2 | Cold Part 1 + Hot Part 2 | 19, 20 |
|  | 3-1 | Cold Part 1 + Hot Part 2 + Hot Part 3 reduced speed | 21 |
|  | 3-2 | Cold Part 1 + Hot Part 2 + Hot Part 3 | 22-24 |

Table S. 3: Weighting factors of the driving cycle phases for the calculation of average PM and PN emissions over the WMTC (based on Euro 5 prescription) and ECE (based on Euro 4 prescription) test cycles, following the prescriptions of Regulation EU 134/2014 (EU, 2014).

| **Vehicle sub-category** | **Weighting factors** | | | **Applicable on vehicles** |
| --- | --- | --- | --- | --- |
|  | **Part 1** | **Part 2** | **Part 3** |  |
| **WMTC** | | | | |
| L1e, L2e, L3e-A1, L5e-A, L6e | 0.5 | 0.5 | - | 1-16, 18, 29,30 |
| L3e-A2/A3 | 0.25 | 0.5 | 0.25 | 17, 19-24 |
| L5e-B, L7e-B | 0.3 | 0.7 | - | 25-28 |
| **ECE** | | | | |
| All | 0.3 | 0.7 | - | All |

The weighting factors of WMTC phases for vehicles of sub-categories L3e, L4e, L5e-A and L7e-A with maximum speed lower than 130 km/h (applicable on vehicles 15-19 of the current study) were revised by EU Regulation 2018/295 (EU, 2018) (0.3 for part 1 and 0.7 for part 2). SPN23 emission levels over the WMTC (average for vehicles 15-19) are 25% lower (due to smaller effect of cold start) when calculated with the revised weighting factors, while the respective reduction in SPN10 emissions is 13%. TPN10 emissions with the revised weighting factors are 18% higher (due to the fact that TPN10 emissions were found to be higher in the second part of the driving cycle, as discussed in section 3.3). Finally, PM emissions are 17% lower when calculated with the revised factors. In all cases, the revision of weighting factors does not bring any changes to the overall trends and conclusions observed in the current study.

# Figures





Figure S.1: Average coefficient of variation (CV) of particulate mass and solid particle number emissions above 23 nm (SPN23) among the test repetitions (WMTC and ECE) of each vehicle.





Figure S.2: Total hydrocarbon (THC) and particulate mass (PM), solid particle number (SPN23) emissions correlation for 2-Stroke mopeds over the WMTC and ECE. Trend line (least square regression applied to WMTC and ECE combined) is presented in each case. Extrapolating these data to Euro 5 THC emissions limit (0.1 g/km), SPN23 levels at the range of 1.5×10^11^ km^-1^ are expected.

# References

EU, 2018. Commission Delegated Regulation (EU) 2018/295. Official journal of the European Union 4.

EU, 2014. Commission Delegated Regulation (EU) No 134/2014. Official journal of the European Union 1–327.

EU, 2013. Regulation (EU) No 168/2013 of the European Parliament and of the Council. Official Journal of the European Union L 60/52.
